# MMP-2 and sTNF-R1 Variability in Patients with Essential Hypertension: 1-Year Follow-Up Study

**DOI:** 10.5402/2012/501894

**Published:** 2012-09-13

**Authors:** Núria Carpena, Esther Roselló-Lletí, Jose R. Calabuig, Estefanía Tarazón, Jose R. González-Juanatey, Luis Martínez-Dolz, Antonio Salvador, Lilian Grigorian, Plácido Orosa, Manuel Portolés, Miguel Rivera

**Affiliations:** ^1^Cardiocirculatory Unit, Research Center, Hospital Universitario y Politécnico La Fe, 46009 Valencia, Spain; ^2^Internal Medicine, Hospital Universitario y Politécnico La Fe, 46009 Valencia, Spain; ^3^Cardiology Unit, Hospital Clínico Universitario de Santiago de Compostela, 15706 Santiago de Compostela, Spain; ^4^Cardiology Unit, Hospital Universitario y Politécnico La Fe, 46009 Valencia, Spain; ^5^Cardiology Unit, Hospital San Francesc de Borja, 46702 Gandía, Spain; ^6^Cell Biology and Pathology Unit, Research Center, Hospital Universitario y Politécnico La Fe, 46009 Valencia, Spain

## Abstract

The aim of this study is to analyze MMP-2 and sTNF-R1 variability, potent predictors of cardiovascular events, in stable hypertensive patients during a 12-month followup. 234 asymptomatic patients (age 60 ± 13, 136 male) out of 252 patients with essential hypertension were followed up. MMP-2 and sTNF-R1 were measured at baseline and after 12 months (stage I). To compare MMP-2 and sTNF-R1 levels over time interval, we used the statistical method of Bland-Altman. MMP-2 and sTNF-R1 reproducibility was good in our patients for the two intervals with a coefficient of reproducibility of 8.2% and 11.3%, respectively. The percentages of patients within 1.96 × standard deviation of the mean were 93.6% and 92.7%. An elevated coefficient of correlation was obtained for MMP-2, basal versus stage I (*r* = 0.55, *P* < 0.0001) and for sTNF-R1 (*r* = 0.75, *P* < 0.0001). There is good stability in MMP-2 and sTNF-R1 levels in a followup study of patients with stable hypertension. As a consequence, assessment of its concentrations may be a useful tool for monitoring the follow-up of these patients. Measured variations in MMP-2 and sTNF-R1 levels, exceeding 8.2% and 11.3%, respectively, may indicate an increase in cardiovascular risk, thus, could be used to optimizing treatment than blood pressure control alone.

## 1. Background

Sustained hypertension (HT) may cause left ventricular hypertrophy (LVH), alterations in cardiac function, and heart failure (HF) [1]. Cardiomyocyte apoptosis has been shown to contribute to myocardial remodeling in response to pressure overload in HT [2, 3].

The soluble tumor necrosis factor receptor 1 (sTNF-R1) represent the classical death signal of the apoptotic process [4, 5]. Overexpression of myocardial TNF-*α* is observed in patients with pressure overload states and cardiac dilation. Evidence supports that circulating sTNF-R1 levels are increased in patients with HF [6–8], being a powerful predictor of mortality in HF [9]. In addition, this receptor is associated with inflammatory disease in HT, it has published that plasma sTNF-R1 was an independent predictor of left ventricular mass index and hypertrophy [10]. 

Changes in the composition of the extracellular matrix (ECM) are known to occur in HT [11, 12], and these may lead to cardiac fibrosis and diastolic dysfunction contributing to the progression of HF and to sudden death [13, 14]. The variations in metalloproteinases (MMPs) expression are important processes of ventricular remodeling in the pathophysiology of HT [15] and may contribute to HF and other cardiac complications in patients with hypertensive heart disease [12, 16]. The serum levels of matrix MMPs increase during chronic HF and metalloproteinase-2 (MMP-2) is related to diastolic dysfunction [17, 18] and to a poor prognosis, being predictor of mortality [19–21].

In a recent study, our group has found a good stability of amino-terminal propeptide of B type natriuretic peptide (NT-proBNP) levels [22], a powerful predictor of mortality in hypertensive patients without HF [23]. Furthermore, we have shown a significant relationship between this natriuretic peptide and inflammatory status, especially with sTNF-R1 [22]. The knowledge of variations in MMP-2 and sTNF-R1 levels is crucial when using these molecules as a tool to monitor the evolution of inflammation activation and collagen remodeling in hypertensive patients. However, there are no studies addressing its variability and stability, and there are no data on the changes in serum MMP-2 and sTNF-R1 levels over time in asymptomatic stable patients with essential HT. This would allow us to know the usefulness of these molecules in the clinical arena.

We hypothesized that inflammatory and fibrosis markers levels may change over time even in patients with clinically stable HT. Therefore, the aims of the present study were to analyze MMP-2 and sTNF-R1 variability during a 12-month followup, in a cohort of stable hypertensive patients.

## 2. Methods

### 2.1. Patients

The study was on 252 Caucasian asymptomatic consecutive out-patients with mild hypertension (mean (SD) age 60 (13) years, 136 male), from 11 participating hospitals. All patients underwent a routine physical examination, electrocardiogram, echo-Doppler study, and laboratory analyses. Physicians using a standardized protocol measured systolic and diastolic blood pressure in the left arm of seated subjects between 08:00 and 11:00 AM, following the recommendations of The American Heart Association[24]. Of the 252 subjects, 234 asymptomatic (they did not refer any symptoms of cardiovascular origin, specifically symptoms of HF) and stable patients (without cardiovascular events [25]) were included in the study (136 with LVH and 98 without LVH, age 60 ± 13 years, 136 male). Eighteen (7%) were excluded during the followup (8 refused to continue, 9 could not be located, and 1 patient had stroke). 

Patients analyzed in this study met these inclusion criteria: a previous diagnosis of hypertension, as defined by the “seventh report of the joint national committee on prevention, detection, evaluation, and treatment of high blood pressure” [24]. Furthermore, exclusion criteria were secondary HT, left ventricular ejection fraction <50, ischemic (medical history, echo-Doppler, troponin T assay) or dilated cardiomyopathy, atrial fibrillation, more than mild valvular disease, acute and chronic liver or renal diseases, immunological diseases, HIV, alcoholism and drug addiction, and any other life-threatening disease.

All patients were on stable medical therapy for at least 2 months before study enrollment with angiotensin II receptor antagonist 50%, diuretics 45%, angiotensin-converting enzyme inhibitors 32%, *β*-blockers 21%, statins 26%, and calcium-channel blockers 19%. There were not statistically significant changes in the different drugs administered during followup. None of the 234 patients finally studied presented cardiovascular events (defined as stroke, myocardial infarction, or cardiovascular death) [25]. Body mass index was calculated as the weight in kilograms divided by height in meters squared, and obesity was defined as body mass index >30 kg/m^2^. Glomerular filtration rate was calculated using the modified diet in renal disease equation [26]. All patients were followed up until the end of the study at month 12, with a two-stage sample collection: basal and 12 months (stage I). All explorations were conducted at each stage. The procedure was approved by the appropriate institutional review boards or ethics review committees of each study center, and the study was conducted in accordance with the guidelines of good clinical practice and with ethical standards for human experimentation established by the Declaration of Helsinki. Every patient signed a written informed consent for their inclusion in the study.

### 2.2. Laboratory Determinations

Venous blood was taken by venipuncture into pyrogen-free vacuum tubes containing EDTA, and serum was obtained by gel clotter tubes, from subjects in sitting position between 8:00 and 11:00 AM. Samples were centrifuged immediately, frozen at −80°C, and only thawed once. Plasma concentrations of MMP-2 and sTNF-R1 were determined at central laboratory by specific commercial sandwich enzyme-linked immunosorbent assay (Hbt human sTNF-R1 ELISA test kit, Hycult Biotechnology, Germany; MMP-2 Human ELISA Kit, Camarillo, CA, USA). The MMP-2 and sTNF-R1 tests have limits of detection of 0.1 ng/mL and 25 pg/mL, respectively. Our intra-assay and interassay coefficients of variation were 5.9 and 6.2% for MMP-2, and 26.5 and 9.1% for sTNF-R1.

### 2.3. Echo-Doppler Study

The examinations were performed using standard systems equipped with 2.5–4 MHz transducers. The echocardiographic examinations were performed using the standard apical and parasternal long axis views were obtained in all echocardiographic studies and analyzed by a computerized system (Eco-Dat; Software Medicina S.A., Madrid, Spain). M-Mode and two-dimensional images, Doppler spectrum and color Doppler were measured and averaged for each Doppler variable. For each patient, four consecutive beats were measured and averaged for each Doppler variable.

Left ventricular ejection fraction (LVEF) was calculated with the area-length method [27]. The E/A ratio was also calculated. Left ventricular mass (LVM) was measured following the Devereux method [28] and indexed for height^2.7^ due to the high prevalence of obese and overweight patients, defining LVH as LVM index >46.7 g/m^2.7^ in women and >49.2 g/m^2.7^ in men [29]. 

### 2.4. Statistical Analysis

Continuous variables are presented as mean ± SD and categorical variables as a number of patients or percentage. Results for each variable were tested for normality using the Kolmogorov Smirnov method. MMP-2 and sTNF-R1 concentrations exhibit a nonnormal distribution and were presented as the median and interquartile range and log transformed (and proved to be normalized) before parametric correlation analysis by Pearson's coefficient. Temporal changes in molecule levels and clinical characteristics were analyzed using the paired student's *t*-test and categorical variable changes were compared using the McNemar test. 

To compare MMP-2 and sTNF-R1 levels over time interval (stage I-basal stage), we used the statistical method of Bland-Altman [30, 31]. In this graphical method the percentage of change in the averages ((molecule stage I-molecule basal)/(average stage I + basal stage)) is plotted against the average of the total molecule measurement. This expression is useful to normalize and compare the data without taking into account the magnitude of the molecule measurement. Based on this approach, the limits of agreement were determined by the mean difference plus or minus the coefficient of reproducibility (CR), where CR was calculated as 1.96 × SD of the percentage of changes. In this case, a high CR indicates poor reproducibility. 

Furthermore, a multivariate linear regression analysis was performed using log transformed MMP-2 and sTNF-R1 as dependent variables and included gender, blood pressure, body mass index, LVMI and known HT duration, and treatment as independent variables. The discrimination of the best model was based on the principle of least mean square and higher R-square. 

A *P value *<0.05 was considered significant for all measures. All statistical analyses were performed using the SPSS 11.5 statistical software package (SPSS Inc, Chicago, IL).

## 3. Results 

The baseline characteristics of the 234 hypertensive patients included in the study in the two stages are shown in Table 1. Significant differences in blood pressure and total cholesterol levels were observed with respect to the basal stage. Body mass index, heart rate, biochemical values, left ventricular mass index and diastolic function variables did not show any statistical change. There were no statistically significant changes between the different drugs administered during the follow-up. The values for MMP-2 were basal stage, 107 (96–122) ng/mL, and stage I, 112 (94–129) ng/mL, *P* < 0.05, and for sTNF-R1 basal stage, 385 (290–541) pg/mL, stage I, 394 (289–576) pg/mL, with no significant differences.

**Figure 1:**
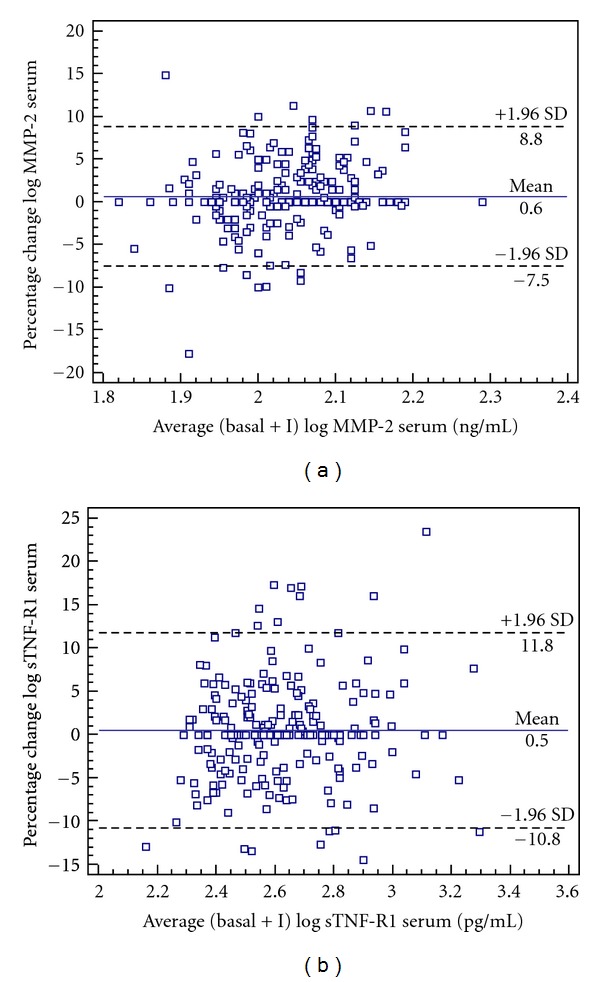
Bland-Altman plots for changes in MMP-2 and sTNF-R1 serum levels in hypertensive patients. Bland-Altman plot showing agreement between the logarithm of MMP-2 levels percentage change against the average of the logarithm of MMP-2 in basal stage + stage I, in the hypertensive patients (a). Bland-Altman plot showing agreement between the logarithm of sTNF-R1 levels percentage change against the average of the logarithm of sTNF-R1 in basal stage + stage I, in the hypertensive patients (b). The solid line represents the mean of the percentage change. The dashed lines define the limits of agreement (standard deviation of percentage of change × 1.96 SD). SD: standard deviation; MMP-2: metalloproteinase-2; sTNF-R1: soluble tumor necrosis factor receptor 1; stage I: 12-month followup.

**Figure 2:**
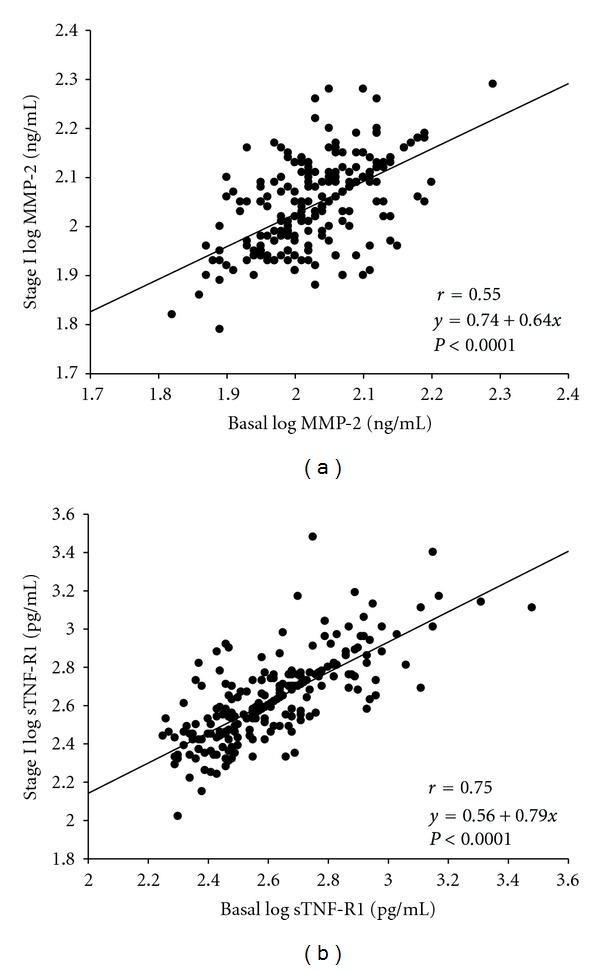
Correlation between log transformed values of MMP-2 (a) and sTNF-R1 (b) in basal stage versus stage I. MMP-2, metalloproteinase-2; sTNF-R1: soluble tumor necrosis factor receptor 1; stage I: 12-month followup.

We obtained good reproducibility for MMP-2 and sTNF-R1 measurements for the whole study comparing stage I-basal stage.  Figure 1 shows the Bland-Altman plots for changes in MMP-2 and sTNF-R1 serum levels in patients with asymptomatic HT, over the interval studied. The percentages of patients within 1.96 SD of the mean were 93.6% and 92.7%, respectively. Furthermore, the values of the mean ± SD percentage change and CR for MMP-2 were 0.6 ± 4.2 with a CR of 8.2% and for sTNF-R1 were 0.5 ± 5.8 with a CR of 11.3%. In addition, when we analyzed the correlation between the two molecules in stage I versus basal stage (Figure 2), we found a significant coefficient of correlation for MMP-2 (*r* = 0.55, *P* < 0.0001) and sTNF-R1 (*r* = 0.75, *P* < 0.0001).

When we correlated MMP-2 with well-established parameters, we found a significant correlation with E/A ratio (*P* < 0.0001), with LVMI (*P* < 0.0001), and with BMI (*P* = 0.005). We also found a correlation between sTNF-R1 and the same parameters, with E/A (*P* < 0.0001), with LVMI (*P* < 0.0001), and with BMI (*P* = 0.016). The correlation between MMP-2 and sTNF-R1 was *P* < 0.0001. When we related both molecules with cholesterol neither MMP-2 nor sTNF-R1 were significantly correlated.

Finally, a multivariate linear regression analysis was used to test the independent predictive power of the related significant variables and treatment on serum log-transformed MMP-2 and sTNF-R1. Neither cholesterol nor blood pressure was independent factors of MMP-2 and sTNF-R1.

## 4. Discussion

The present study shows in a homogeneous and representative group of patients with clinically and functionally stable hypertension a good stability of MMP-2 and sTNF-R1 levels. This is the first study to monitor changes in serum MMP-2 and sTNF-R1 concentration in a 12-month followup in patients with essential HT.

Hypertensive heart disease is a progressive condition in which the compensatory LVH leads to myocardial remodeling, characterized by fibrosis and decrease in the number of cardiomyocytes. It has been suggested that alterations of the collagen turnover and apoptosis may be one of the mechanisms involved in the genesis of diastolic dysfunction of hypertensive origin [32]. Cardiomyocyte apoptosis has been shown to be abnormally stimulated in the hypertrophied heart of patients with essential HT [33] and moderate cardiomyocytes loss in long-term systemic HT with no clinical evidence of HF [34].

Because of the detrimental effects that cardiomyocyte apoptosis and fibrosis may exert in hypertensive heart disease, to recognize and prevent or limit the magnitude of this phenomenon may be relevant in both assessing and modifying the clinical outcome of patients with arterial HT. It would be of interest to monitor such processes of apoptosis, with sTNF-R1, and abnormal ECM metabolism, with MMP-2, in hypertensive patients by using these molecules variability as a tool to monitor the evolution in HT patients and to achieve an earlier prognosis or prevention of hypertension-induced HF. MMP-2 and sTNF-R1 are potent predictors of cardiovascular events and mortality in HF patients, and these biochemical markers used in conjunction with other established markers as NT-proBNP, that is, a powerful predictor of mortality in HT [22], may also help to identify patients with no clinical evidence of HT, and provide information about the need for changes in treatment during different stages of the disease, and potentially it could provide valuable biochemical data for the specialist.

The main clinical consequence of this study is the establishment of a MMP-2 and sTNF-R1 percentage change, from which we can monitor the progress of these patients. Thus, we suggest that MMP-2 measured variations, with a coefficient of reproducibility (>1.96 SD percentage change) above 8.2% and for sTNF-R1, values over 11.3% may be considered of potential clinical value when monitoring hypertensive patients. Although there are differences in the average of MMP-2 levels compared with stage I versus basal stage, with the Bland-Altman analysis we can observe that this molecule is one of the most stable and has the lowest coefficient of reproducibility, indicating that these variations in the average do not affect its stability as a marker of in hypertensive disease development. Furthermore, good correlation was obtained between MMP-2 and sTNF-R1 concentrations at the two stages over the entire study, the correlation coefficients being higher for the sTNF-R1 levels of hypertensive patients.

In our 234 patients with clinically and functionally stable HT there were neither cardiovascular events nor differences in ventricular function. Moreover, we found significant differences for values of blood pressure and total cholesterol of these variables decreasing over time probably as a consequence of the treatment. At first we might think that these changes in blood pressure values may affect the molecules concentrations during followup and thus alter its intrinsic variability and limit the purpose of the study. However, through the simple correlations and the multivariate linear regression analysis carried out we can confirm that neither blood pressure nor total cholesterol levels were independent predictors of these molecule levels in hypertensive patients, as other authors have pointed out [10, 35, 36]. Therefore MMP-2 and sTNF-R1 variability could not be attributed to their changes over time.

One limitation of this study is that our patients were on medication and it is known that these molecule values could be affected by treatment with diuretics, angiotensin-converting enzyme inhibitors, angiotensin II receptor blockers, or beta-blockers [37–40]. Nevertheless, this circumstance makes it easier to extrapolate our data to the clinical practice. On the other hand, we have to admit that a larger group of patients would have provided additional information. However, the strict inclusion-exclusion criteria give our results greater value. 

An important consideration is that we selected patients with clinically stable HT without clinical or functional changes, but we cannot rule out the possibility of subtle changes in neurohormonal and immunology systems that might potentially influence the variability of the molecule levels. In some studies, the increase of apoptotic markers was associated with comparable changes of other cytokine levels, indicating that the activation of this system is also associated with autoimmune-inflammatory reactions [41, 42]. However, we think that because of this, our data are more useful for judging the clinical variations in these molecule levels, and they have evident practical application. 

Another potential limitation is that although echocardiography-standardized techniques have been shown to be a more sensitive tool for detecting LVH than for electrocardiographic measurements, the variability of this technique is higher than the variability using magnetic resonance imaging. However, in this study a specialized, blinded, single cardiologist performed the echocardiographic analysis to minimize variability of the measurements. Moreover, it would have been interesting to get the filling pressures, but the methodology used in our asymptomatic patients did not include this measure. 

## 5. Conclusions

This study shows that there is good stability in MMP-2 and sTNF-R1 levels in a 12-month follow-up study of asymptomatic patients with clinically and functionally stable hypertension. Measured variations in MMP-2 exceeding 8.2% in a 12-month followup and for sTNF-R1, values over 11.3% may indicate an increase in cardiovascular risk. The low variability of these molecules suggest that their levels could be used to monitor hypertensive patients and optimizing medical treatment rather than blood pressure control alone.

## Figures and Tables

**Table 1:** Clinical characteristics of patients with essential hypertension over the entire study (*n* = 234): basal, stage I = at 12 months.

	Basal (*n* = 234)	Stage I (*n* = 234)
Age (years)	60 ± 13	61 ± 13
Gender (% male)	54	54
BMI (kg/m^2^)	30 ± 4	30 ± 4
SBP (mmHg)	149 ± 20	142 ± 11^b^
DBP (mmHg)	87 ± 11	84 ± 11^b^
PP (mmHg)	62 ± 18	58 ± 17^b^
GFR (mL/min/1.73 m^2^)	93 ± 39	95 ± 25
Total cholesterol (mg/dL)	212 ± 37	206 ± 33^a^
Urea (mmol/L)	41 ± 11	42 ± 14
Sodium (mmol/L)	141 ± 4	141 ± 4
Potassium (mmol/L)	4.2 ± 0.4	4.3 ± 0.4
EF (%)	59 ± 5	59 ± 5
E/A ratio (m/s)	0.91 ± 0.25	0.94 ± 0.26
LVMI (g/m^2.7^)	53 ± 17	53 ± 17

Results are shown as mean ± SD or percentage of subjects. BMI: body mass index; SBP: systolic blood pressure; DBP: diastolic blood pressure; PP: pulse pressure; GFR: glomerular filtration rate; EF: ejection fraction; E: maximal early mitral valve inflow; A: maximal late mitral valve inflow; LVMI: left ventricular mass index. ^a^ *P* < 0.05; ^b^ *P* < 0.01.
